# Molecular Evidence of Human Fasciolosis Due to *Fasciola gigantica* in Iran: A Case Report

**Published:** 2018-05

**Authors:** Mohammad Bagher ROKNI, Arezoo BOZORGOMID, Peyman HEYDARIAN, Mojgan ARYAEIPOUR

**Affiliations:** 1. Dept. of Medical Parasitology and Mycology, School of Public Health, Tehran University of Medical Sciences, Tehran, Iran; 2. Dept. of Microbiolgy, Asadabad School of Medical Sciences, Asadabad, Iran; 3. Dept. of Medical Parasitology and Mycology, School of Medicine, Qazvin University of Medical Sciences, Qazvin, Iran

**Keywords:** *Fasciola gigantica*, PCR, ELISA, Diagnosis, Iran

## Abstract

Fascioliasis is a foodborne zoonotic disease caused by the two parasite species *Fasciola hepatica* and *F. gigantica*. In spite of the presence of both species of *Fasciola* in the livestock, to our knowledge, to date, no cases of human *F. gigantica* infection have been reported in Iran officially. Here, we report such a case in a 25 yr old woman referred to The Department of Medical Parasitology and Mycology, School of Public Health, Tehran University of Medical Sciences, Tehran, Iran in 2016. CT imaging and MRCP revealed an ill-defined lesion of segments of liver. Specific ELISA produced a positive result besides detecting egg of the parasite via stool exam. The identification of parasite species was performed by the DNA extracted from the eggs and sequencing ITS-1, in addition to comparison to GenBank retrieved sequences, using the BLAST search tool. The sample showed 100% identity with *F. gigantica*. She was treated for fasciolosis with a single dose of Egaten® 10 mg/kg with positive response. This is the first case of human fasciolosis due to *F. gigantica* reported in Iran.

## Introduction

Fascioliasis is primarily an infection of livestock such as sheep and cattle and caused by two parasite species of the genus *Fasciola* as *F. hepatica* and *F. gigantica*. Humans become accidental hosts through eating watercress or other fresh aquatic vegetation and by drinking contaminated water containing viable metacercariae of the parasite (1).

The life cycle of *Fasciola* spp. is dependent on the presence of a snail as an intermediate host. *Lymnaea truncatula*, the main intermediate host snail for *F. hepatica*, is mainly found in temperate regions. In contrast, *F. gigantica* is mainly found in tropical and sub-tropical regions, where *L. auricularia* and *L. gedrosiana* species have been reported as the main intermediate hosts (2, 3).

The diagnosis of fascioliasis can be established by the observation of the parasite’s egg in stool, although serological tests has a higher sensitivity and specificity in the acute phase of disease, even before the parasite eggs can be identified in the stool (4).

In Iran, the disease is most frequently seen in Northern region especially in Guilan Province (5, 6) and sporadic cases have been rarely reported from other regiones (7).

In spite of the presence of both species of *Fasciola* in the livestock, to our knowledge, to date, no cases of human *F. gigantica* have been reported in country.

Here, for the first time, we report parasitological observations and molecular evidence of human *F. gigantica* infection in a woman in Iran.

## Case Report

**Fig. 1:**
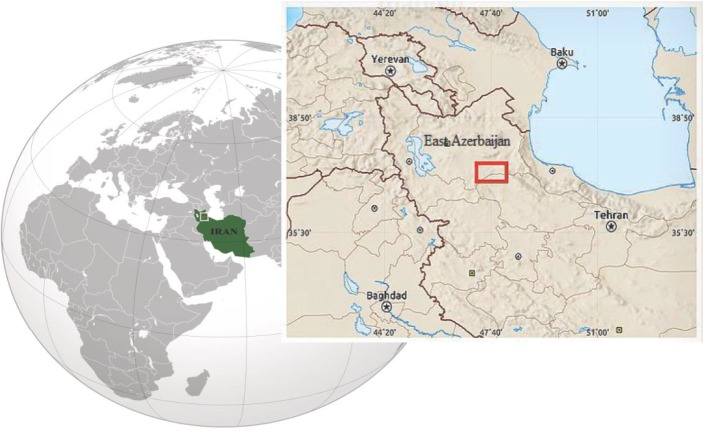
Location of Mianeh District, Mianeh, East Azerbaijan Province, Iran

In 2016, a 25-yr-old woman complained of fever and abdominal pain. She was living in a small village in Mianeh, East Azerbaijan Province, Iran (Fig. 1).

The latitude for Mianeh is: 37.426434 and the longitude is 47.724111. The patient declared a regular picking and eating local watercress in the rural area. She was admitted to a local hospital and was treated symptomatically. Despite treatment, fever, jaundice, right upper quadrant abdominal pain and loss of appetite continued for 6 months. Informed consent was taken from the patient.

Initial laboratory findings were as follows: white blood cells 4.3×10^3^ μL, platelets 245 ×10^3^ μL, hemoglobin 12.3 g/dL, hematocrit 37.9%. Peripheral blood smear revealed eosinophilia as high as 36% of the white blood cells. Stool examination was negative for ova/cysts. Liver function tests showed elevated liver enzymes (aspartate transaminase 37 [normal < 31] U/L, alanine transaminase 63 [normal < 32] U/L, alkaline phosphatase 458 [normal 98–279] U/L and a normal total bilirubin.

Abdominal ultrasonography showed splenomegaly (140 mm). A magnetic resonance cholangiopancreatography (MRCP) revealed an ill-defined lesion of 96×53 mm at 4^th^ and 8^th^ segments of liver with dilation of intrahepatic bile ducts inside the lesion and splenomegaly was seen. The initial pre-treatment CT imaging revealed a hepatosplenomegaly with regional lymphadenopathy and several subcapsular lesions in both liver lobes.

In the search for a potential malignancy, liver biopsy was performed. Section from liver tissue showed focal lobular necrosis with peripheral palisading spindle-shape epithelioid cells; surrounded by moderate inflammatory cells mainly eosinophils and plasma cells beside a few lymphocytes. Other parts of liver tissue showed mild infiltration of a few eosinophils, lymphocytes and few plasma cells without piecemeal necrosis or bile duct damage, which led to the suspicion of a parasitic infection (toxocariasis, capillariasis or strongyloidiasis).

**Fig. 2:**
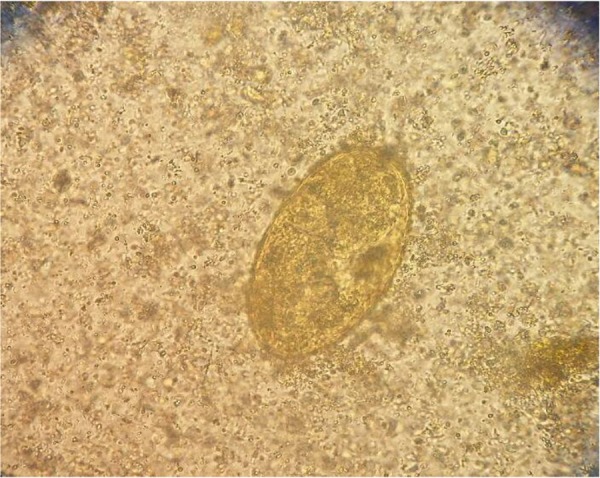
Egg of *Fasciola* from patient’s fece sample under the microscope (40X) (Original figure)

On May 24, 2016, the patient was referred to the Department of Medical Parasitology and Mycology of Tehran University of Medical Sciences, Tehran, Iran. After reviewing the clinical / para-clinical findings and interviewing, fascioliasis was suspected. ELISA test was performed as previously described (8). Antibodies specific to *Fasciola* ES antigens were detected in the serum sample. Furthermore, the formalin ether concentration technique showed the presence of *F. gigantica* eggs (135 μm-80 μm) in feces (Fig. 2).

Egaten treatment was started at the dose of 10 mg/kg. The drug was given as a single oral dose. Three months after treatment, the patient was examined again, fecal examination was negative for *F. gigantica* eggs, and IgG titers decreased but remained positive during 9 subsequent months. Blood examinations revealed an improvement in the liver function tests with a decrease in blood eosinophil counts (5%)

### Molecular analysis

**Fig. 3:**
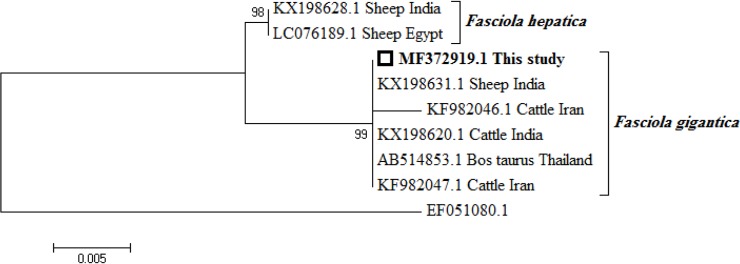
Phylogenetic tree based on 700 bp nucleotides of ITS1 gene showing relationships between *Fasciola hepatica*, *Fasciola gigantica* and our specimen using maximum likelihood method. *Fascioloides magna* (AN: EF051080) was used as outgroup. Numbers at the nodes indicate percentage of bootstrap support obtained in 1,000 replicates. The scale bar indicates the p-distance of the branches

In order to provide genetic diagnosis, the stool sample was washed twice with phosphate buffered saline (PBS) to remove the ethanol. The egg walls were mechanically disrupted using glass beads (0.45–0.52 mm diameter) and by freezing and heating (−70 °C for 5 min and 90 °C for 5 min). Total genomic DNA was extracted, using a commercial kit (QIAamp DNA Stool Mini Kit; Qiagen GmbH, Hilden, Germany) according to the manufacturer’s instructions. ITS-1 fragment (about 700 bp) was amplified by PCR using a set of 5’- ACCGGTGCTGAGAAGACG -3’ and 5’- CGACGTACGTGCAGTCCA -3’ as forward and reverse primers, respectively, following the protocol previously described (9). The negative control was a reaction mixture of distilled, without the DNA template. . The PCR products were separated in 1.5% agarose gel using Simply Safe (Eurx, Cat. No. E4600-01). A phylogenetic tree was constructed, based on the ITS-1 gene sequences to show the relationships between the available sequences of *F. hepatica*, *F. gigantica* in the GenBank, and sequence of our sample (700 bp), using the MEGA 6 software (Fig. 3) (10).

## Discussion

While fascioliasis is a well-known human parasite, it is difficult to diagnose the disease in non-endemic regions, due to low incidence of the disease and also limited experience with fascioliasis.

In our case, absence of eggs in the initial stool examination probably explained due that the patient was in an acute phase of infection and did not pass eggs during this phase. Stool exam is the best method (Gold standard) for the diagnosis of the disease (1). Nevertheless, worm eggs appear in the feces between 3–4 months after the infection (1). Even during the chronic phase of the infection, more than one stool specimen may need to be examined to find eggs, especially in people with light to moderate infections (11).

Among the laboratory finding, significant eosinophilia might be the best considered blood test, especially during the acute phase of the infection, when the young parasite migrates through the liver of the patient. Immunologic techniques, such as ELISA are the main diagnostic tests since anti-*Fasciola* antibodies in the serum can be detected as early as two weeks after the infection (11). They have 100% sensitivity and 97.8% specificity for diagnosis of fascioliasis (12). Computerized tomography (CT) scan, ultrasonography and MRI (magnetic resonance imaging) are useful techniques for the diagnosis of fascioliasis (13).

The clinical presentation of fascioliasis is often vague and unspecific with a wide variety of disorders. Our case had history of right upper quadrant abdominal pain, fever, jaundice. In addition, the laboratory examinations indicated elevated liver enzymes and eosinophilia that could make one suspect fascioliasis.

History of watercress consumption may be helpful for the exact diagnosis. In our case, a history of ingesting local watercress was noted. The main source in the transmission of this infection is the aquatic plants and contaminated water and reservoir animals plays more important role (1). Therefore, she had a high risk for the disease.

Diagnosis of fascioliasis on the bile duct was not done before the surgery in some cases (14, 15). Although liver biopsy may be an important part of patient assessment, it is an invasive method with a relatively high risk of complications. In our case, liver biopsy was recommended for further evaluation. Non-invasive techniques such as parasitological and immunologic approaches are widely employed for the diagnosis of human and animal fasciolosis (1).

To our knowledge, the present case report is the first molecular evidence of human *F. gigantica* infection in Iran. It has been previously postulated that *F. gigantica* might be the most common species infecting humans in northern Iran (16). However, finding the proper causative agent of the human fasciolosis, based on the present facilities is somehow very difficult. Our molecular study revealed that the parasites from the different geographical regions have close phylogenetic associations. Also little or no intraspecific variation was detected between parasites from different hosts.

## Conclusion

Considering that more documents are needed to present the human fasciolosis due to *F. gignatica* in Iran, further studies of such structure are required to clarify this issue in this important region of fasciolosis.

## Ethical considerations

Ethical issues (Including plagiarism, informed consent, misconduct, data fabrication and/or falsification, double publication and/or submission, redundancy, etc.) have been completely observed by the authors.
